# Characterization and Plasma Measurement of the WE-14 Peptide in Patients with Pheochromocytoma

**DOI:** 10.1371/journal.pone.0088698

**Published:** 2014-02-11

**Authors:** Johann Guillemot, Marlène Guérin, Erwan Thouënnon, Maité Montéro-Hadjadje, Jérôme Leprince, Hervé Lefebvre, Marc Klein, Mihaela Muresan, Youssef Anouar, Laurent Yon

**Affiliations:** 1 Institut National de la Santé et de la Recherche Médicale (INSERM), U982, Mont-Saint-Aignan, France; 2 Normandy University, Normandy, France; 3 Rouen University, Laboratory of Neuronal and Neuroendocrine Differentiation and Communication, Institute for Research and Innovation in Biomedicine (IRIB), Mont-Saint-Aignan, France; 4 Laboratory of Biochemical Neuroendocrinology, Clinical Research Institute of Montreal, Montreal, Quebec, Canada; 5 Department of Endocrinology, Diabetes and Metabolic Diseases, Rouen University Hospital, Bois-Guillaume, France; 6 Department of Endocrinology, Hôpital de Brabois, University of Nancy, Nancy, France; 7 Unit of Endocrinology, Hôpital Notre-Dame de Bon Secours, Metz, France; UPR 3212 CNRS -Université de Strasbourg, France

## Abstract

Granins and their derived peptides are valuable circulating biological markers of neuroendocrine tumors. The aim of the present study was to investigate the tumoral chromogranin A (CgA)-derived peptide WE-14 and the potential advantage to combine plasma WE-14 detection with the EM66 assay and the existing current CgA assay for the diagnosis of pheochromocytoma. Compared to healthy volunteers, plasma WE-14 levels were 5.4-fold higher in patients with pheochromocytoma, but returned to normal values after surgical resection of the tumor. Determination of plasma CgA and EM66 concentrations in the same group of patients revealed that the test assays for these markers had an overall 84% diagnostic sensitivity, which is identical to that determined for WE-14. However, we found that WE-14 measurement improved the diagnostic sensitivity when combined with the results of CgA or EM66 assays. By combining the results of the three assays, the sensitivity for the diagnosis of pheochromocytoma was increased to 95%. In fact, the combination of WE-14 with either CgA or EM66 test assays achieved 100% sensitivity for the diagnosis of paragangliomas and sporadic or malignant pheochromocytomas if taken separately to account for the heterogeneity of the tumor. These data indicate that WE-14 is produced in pheochromocytoma and secreted into the general circulation, and that elevated plasma WE-14 levels are correlated with the occurrence of this chromaffin cell tumor. In addition, in association with other biological markers, such as CgA and/or EM66, WE-14 measurement systematically improves the diagnostic sensitivity for pheochromocytoma. These findings support the notion that granin-processing products may represent complementary tools for the diagnosis of neuroendocrine tumors.

## Introduction

Chromogranins/secretogranins or granins (Cgs) represent a family of secretory proteins that occur in large dense-core vesicles of endocrine, neuroendocrine and neuronal cells [1], [2]. Members of the Cg family include chromogranin A (CgA), chromogranin B (CgB), secretogranin II (SgII), SgIII (or 1B1075), SgIV (or HISL-19), SgV (or 7B2), SgVI (or NESP55), SgVII (VGF) and Pro-SAAS [3]. The primary amino acid sequence of Cgs is characterized by the abundance of acidic residues and the existence of several pairs of consecutive basic residues forming potential cleavage sites for endopeptidases. As a result, granins serve as precursor proteins that can be processed by proprotein convertases (PCs) generating a variety of peptides [4], [5]. Thus, post-translational processing of CgA gives rise to vasostatin I and II, chromofungin, chromacin, pancreastatin, catestatin, parastatin, WE-14 and EL35 peptides. The proteolytic cleavage of SgII generates secretoneurin (SN), EM66 and manserin. Their ubiquitous distribution in endocrine and neuroendocrine tissues and their co-secretion with resident peptide hormones and biogenic amines, make granins and their derived peptides useful markers of secretion from neuroendocrine cells and neoplasms [6]. Numerous studies have documented the clinical value of detecting granins in tissues and measuring their circulating levels [7]. In particular, measurement of CgA levels in plasma can be used to diagnose or monitor the progression of neuroendocrine tumors [8]. However, CgA levels may also be elevated in patients with hyperplasia [9] and may therefore not be reliable for distinguishing neuroendocrine hyperplasia from adenoma or carcinoma. In addition, CgA measurement showed a low sensitivity in certain neuroendocrine tumors such as insulinomas, pituitary adenomas and medullary thyroid carcinomas [10], [11]. Thus, measurement of other Cgs or Cg-derived peptides may be helpful for the diagnosis of different neuroendocrine tumors. Indeed, it has been reported that the CgA-derived peptide vasostatin I may help to distinguish between metastatic deposits originating from ileum or lung carcinoid primary tumors [12], and that plasma levels of GAWK and CCB, two CgB-derived peptides, are elevated in patients with pancreatic islet-cell tumors [13], [14] or with bronchial tumors [15]. Similarly, high concentrations of SgII have been found in ganglioneuromas and neuroblastomas [16], while high plasma SN concentrations are associated with several neuroendocrine tumors [17] and with progression of neuroendocrine prostatic carcinomas [18].

Pheochromocytomas are rare catecholamine-producing tumors originating from chromaffin tissues at adrenal and extra-adrenal locations (the latter referred to as paragangliomas). Most of these neuroendocrine tumors occur sporadically, but the proportion of sporadic pheochromocytomas presenting genetic mutations that was initially estimated to about 24% [19] may actually reach 30% or more [20]. The latest gene mutation discoveries brought to 11 the number of genes playing an important role in the pathogenesis of pheochromocytomas. These genes include RET, VHL, NF1, SDHA, SDHB, SDHC, SDHD, SDHAF2, TMEM127, MAX and HIF2α [21], [22]. The malignancy rate of pheochromocytomas varies considerably from less than 10% to up to 40% depending on the location of the primary tumor and the underlying germline mutation [23]. The malignant behaviour of these tumors remains poorly understood and there is a need for improved predictors of malignancy [24]. Unlike benign tumors that can be diagnosed and surgically treated, malignant pheochromocytoma, which is currently uncurable, cannot be reliably identified on the basis of biochemical or histological features. Currently, malignancy of pheochromocytomas can be diagnosed only after metastasis appearance.

CgA is currently the only granin whose measurement is routinely used by clinicians for the diagnosis of neuroendocrine tumors. Although several studies have shown that CgA is a reliable sensitive marker for the diagnosis of pheochromocytoma, its diagnostic specificity is quite poor. Previously we have reported the presence of the 66 amino-acid peptide (SgII_187-252_) named EM66 [25], which is derived from SgII, in chromaffin cells of the rat, bovine and human adrenals [26]–[28]. We additionally found that EM66 levels are significantly increased in the plasma of patients with pheochromocytomas [29]. We also demonstrated that low tissue concentrations of the peptide are associated with malignant differentiation of these neoplasms [30], [31]. Our data suggested that EM66 could potentially be a new diagnostic and prognostic marker of pheochromocytomas, and that, besides CgA, other granin-derived peptides, such as EM66, could represent valuable supplementary markers for the management and follow-up of these tumors.

In addition to EM66, we have also reported that WE-14, a 14 amino-acid peptide derived from the proteolytic processing of CgA (CgA_324-337_) occurs in normal and tumoral (*i.e.* pheochromocytoma) human adrenochromaffin tissues [32] and that this peptide is secreted from pheochromocytes in primary culture [33]. WE-14 was first isolated from ileal carcinoid tumor and pheochromocytoma tissues [34], [35] but was shown later to be widely distributed in multiple neuroendocrine tissues and tumors [36]–[38]. In the present study, we characterized WE-14 in pheochromocytoma and showed that plasma WE-14 measurement, when used in combination with CgA and EM66, represents a potential new tool for the diagnosis of the different subtypes of this neuroendocrine neoplasm.

## Materials and Methods

### Patients and plasma collection

Plasma samples of control subjects were obtained from a group of 21 healthy volunteers including 9 women (mean age 39.7±13.7 year, range 23–60 year) and 12 men (mean age 44.7±14.2 year, range 26–65 year), for WE-14 and EM66 measurements. Plasma samples of patients were obtained from a group of 37 subjects with histologically-proven pheochromocytoma (32 benign and 5 malignant) including 23 women (mean age 43.9±14.6 year, range 21–68 year) and 14 men (mean age 42.75±17.9 year, range 17–62 year). Among the tumors, 26 were pheochromocytomas and 11 were paragangliomas as specified above. Twenty-four tumors were apparently sporadic, whereas 3 tumors had a RET mutation, 2 a NF1 mutation, 3 a SDHB mutation, 4 a SDHD mutation and 1 a VHL mutation. Of note, the seven patients with a SDH mutation had a paraganglioma and the five malignant tumors are of adrenomedullary origin (*i.e.* pheochromocytoma). Plasma samples of healthy volunteers were provided by the Rouen University Hospital Center and those of patients were provided by the Rouen, Nancy and Lausanne University Hospital Centers. After collection, plasma samples were kept frozen at –80°C. The diagnosis of benign pheochromocytoma was based on the absence of histological criteria of malignancy and tumor recurrence or metastatic diffusion during a follow-up of at least 2 years. Malignancy was established on the basis of the presence of at least one metastasis. All samples were obtained according to the requirements of french bioethic laws (2004–800/801 of August 6 2004, 2007–1220 of August 10 2007). The protocols for sample collection and the experimental procedures were approved by the Comité de Protection des Personnes Nord-Ouest I, the Nancy Regional Bioethics Committee (approval number DC-2008-459, 10/10/2008) and the Commission d’Ethique of Lausanne [29]. Written informed consent was obtained from all the healthy volunteers and patients with pheochromocytomas and from the next of kin for the minor patients.

### Peptide synthesis

Human EM66 (SgII_187–252_), human WE-14 (CgA_324–337_) and its N-terminally tyrosylated variant, [Tyr^0^]WE-14, were synthesized by the solid-phase methodology as previously described [39]. The purified peptides were characterized by mass spectrometry.

### Preparation of plasma samples

For reversed-phase HPLC analysis and WE-14 radioimmunoassay (RIA), plasma samples collected from healthy volunteers and pheochromocytoma patients were either kept frozen before prepurification for subsequent HPLC analysis, or dried by vacuum centrifugation and kept at room temperature for further RIA quantification of WE-14 concentrations.

### Prepurification of plasma samples

Plasma samples were loaded onto a Sep-Pak C_18_ cartridge (Water Corps, St-Quentin en Yvelines, France) equilibrated with a solution of 0.1% trifluoroacetic acid (TFA) in water. Bound materials were eluted from the cartridge with a solution of acetonitrile/water/TFA (59.9:40:0.1, vol/vol/vol), dried by vacuum centrifugation and kept at room temperature until chromatographic analysis.

### HPLC analysis

Plasma samples were reconstituted in 1 ml of 0.1% TFA in water, centrifuged at 21,000 *g* (10 min; 4°C), and injected onto a 4.6×250 mm Vydac 218TP54 C_18_ column equilibrated with a solution of acetonitrile/water/TFA (9.9:90:0.1, vol/vol/vol) at a flow rate of 1 ml/min. The concentration of acetonitrile in the eluting solvent was raised to 60% over 25 min using a linear gradient. HPLC standard consisted of 1 µg synthetic human WE-14. Fractions of 0.5 ml were collected, evaporated and kept dry until WE-14 RIA.

### Immunoassays

The concentrations of WE-14 and EM66 in plasma samples were measured by RIA as previously described [26], [32]. Synthetic [Tyr^0^]WE-14 or EM66 peptides were iodinated by the chloramine-T method and separated from free iodine on Sep-Pak C_18_ cartridges using a step gradient of acetonitrile (20–60% and 20–40%, respectively) in 0.1% TFA. The RIA were performed in veronal buffer (pH 7.4) supplemented with 0.4% bovine serum albumin (BSA, Roche Diagnostics, Mannheim, Germany) and 0.1% Triton X-100. Dried samples were reconstituted in the RIA buffer. WE-14 or EM66 antisera (code number 333–1506 and 736–1806, respectively, see [26], [32] for more details) used at a final dilution of 1∶100,000, were incubated with 7,000 cpm of tracer/tube in the presence of graded concentrations of standard (synthetic WE-14 or EM66), plasma samples, or HPLC fractions. After 2-day incubation at 4°C, the antibody-bound fraction was immunoprecipitated by the addition of 100 µl of 1% goat anti-rabbit γ-globulins and 1 ml of 20% polyethyleneglycol 8000. After 20 min of incubation at room temperature, the mixture was centrifuged (5,000 *g*, 30 min, 4°C) and the pellet containing the bound fraction was counted on a gamma-counter (LKB, Wallack, Rockville, MD). The standard curves were set up with concentrations of peptides ranging from 5 to 10,000 pg/tube. The accuracy of the assay of WE-14 was evaluated by adding the peptide at 3 different concentrations (2, 5 and 10 ng/ml) to plasma samples. The recovery for WE-14 was 92.8–109.3%. Interassay coefficients of variation (CV) were 2.67–4.10% and intraassay CV were 1.63–3.12%. The accuracy of the EM66 assay has been previously reported [29].

Concentrations of CgA were determined using a commercial kit based on an immunoenzymatic sandwich methodology. The concentrations were measured in duplicate directly from 50 µl plasma samples following the manufacturer recommendations (DAKO CgA ELISA kit ; Dako A/S, Copenhagen, Denmark). CgA concentrations were expressed as U/l.

### Data analysis

Data are reported as median (min-max). Several nonparametric statistical methods were used such as Mann–Whitney U test and Kruskal–Wallis test. Probability values less than 0.05 were considered significant. The Spearman's test was performed to analyze the correlations between plasma levels of WE-14 and CgA, WE-14 and EM66 or EM66 and CgA and between tumor size and CgA, WE-14 or EM66 plasma levels. Data were analyzed with the Prism program (GraphPad Software, San Diego, CA).

## Results

### Characterization of WE-14-immunoreactivity in plasma of healthy volunteers and patients with pheochromocytoma

**Figure 1:**
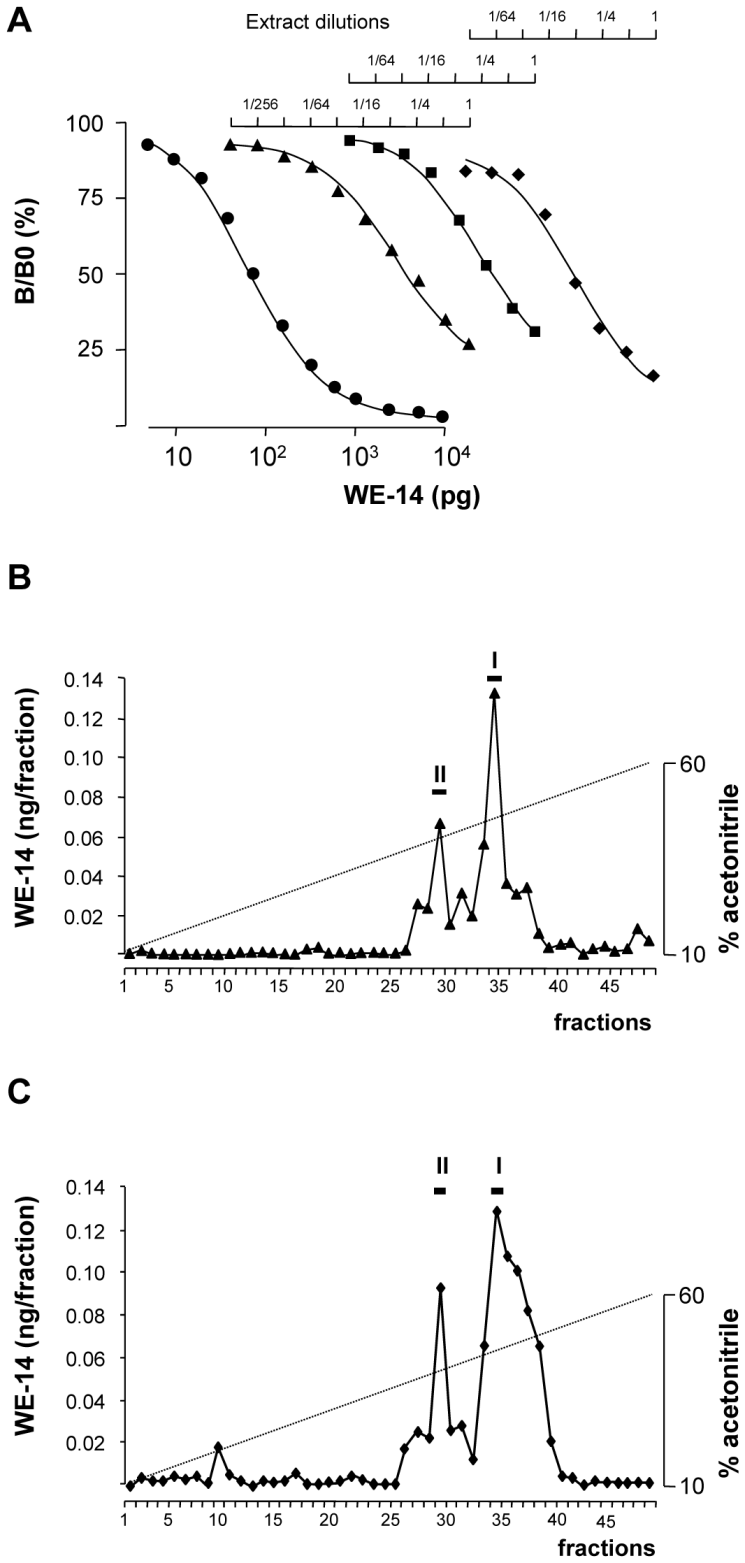
Characterization of WE-14 in plasma. (**A**) Semilogarithmic plots comparing competitive inhibition of antibody-bound ^125^I-labeled Tyr_0_-WE-14 by synthetic human WE-14 (•) and serial dilutions of plasma samples from healthy volunteer (▴), post-operative (▪) and preoperative (♦) patients with benign pheochromocytoma. (**B, C**) Reversed-phase HPLC analysis of WE-14 immunoreactivity in plasma samples from a healthy volunteer (B) and a patient with benign pheochromocytoma (C). The *bars* above the peaks indicate the elution position of synthetic human WE-14 (peak I) and its oxidized form (peak II) chromatographed the same day as the extracts. The *dashed line* shows the concentration of acetonitrile in the eluting solvent.

Serial dilutions of plasma samples from healthy volunteers and preoperative and postoperative patients with benign pheochromocytoma generated displacement curves that were parallel to that obtained with synthetic WE-14 (Figure 1A). Biochemical characterization of WE-14 immunoreactivity in plasma of healthy volunteers (Figure 1B) and patients with benign pheochromocytoma (Figure 1C) showed the occurrence of two WE-14-immunoreactive peaks (noted I and II), which co-eluted with those obtained with the synthetic peptide. Consistent with our previous report [32], peak I corresponded to the reduced form and peak II corresponded to the oxidized form of WE-14.

### Plasma levels of WE-14 in healthy volunteers and patients with pheochromocytoma

**Figure 2:**
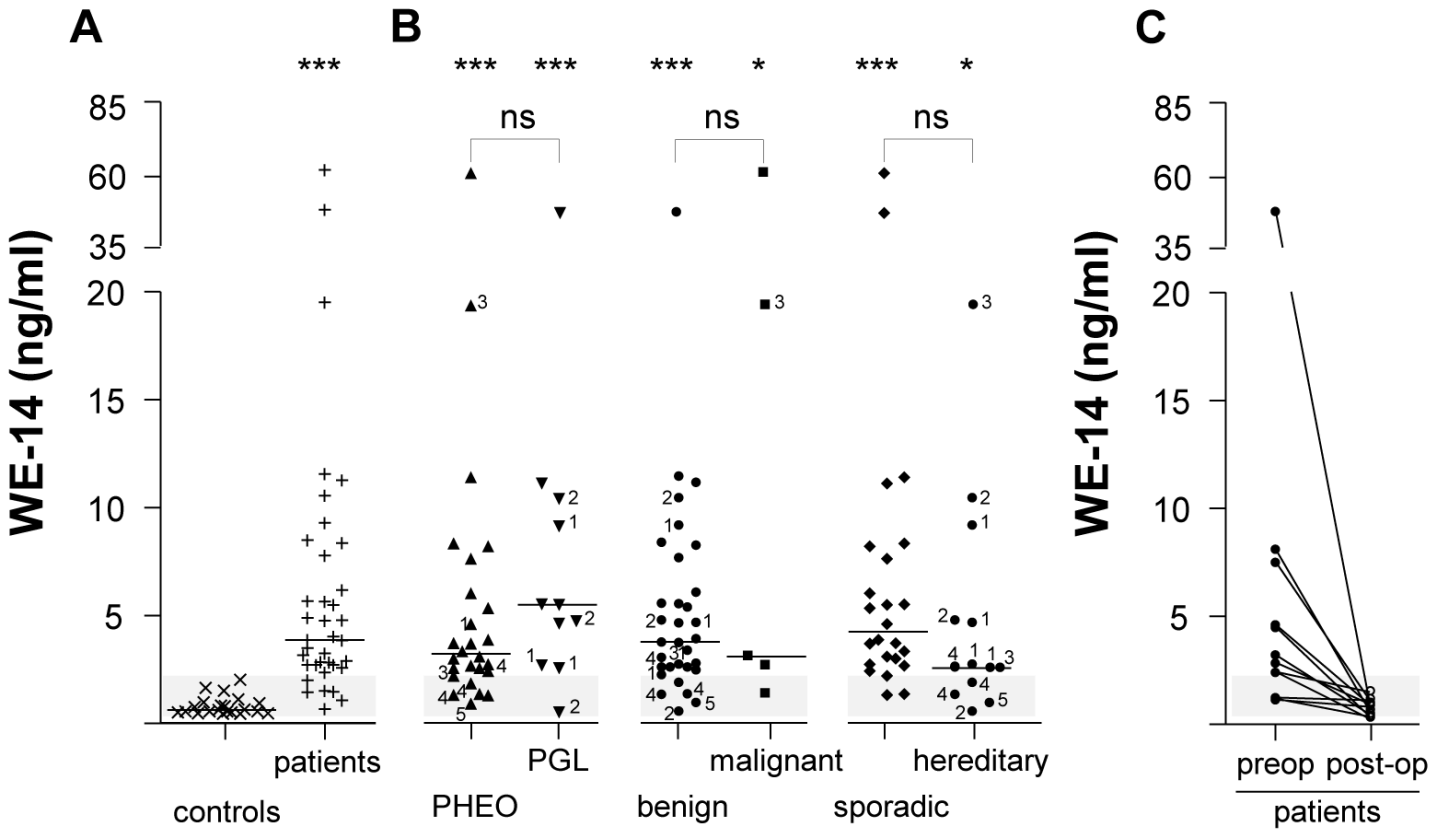
WE-14 levels in the plasma of patients with pheochromocytoma. (A) Scattergram of WE-14 concentrations in plasma samples of healthy volunteers (controls, ×, n = 21), and patients with pheochromocytoma (+, n = 37). (**B**) Distribution of WE-14 preoperative concentrations in patients reported in (A), depending on the adrenal (PHEO, ▴, n = 26) or extra-adrenal (PGL, ▾, n =  11) location of the tumor, the benign (•, n = 32) or malignant (▪, n = 5), and the sporadic (♦, n = 24) or hereditary (•, n = 13) nature of the neoplasms. (C) Distribution of WE-14 levels (n = 12) in preoperative (•, preop) and postoperative patients for which the tumor was resected (○, postop). The *bars* represent the median value for each group. The *grey zone* corresponds to the distribution of control values and indicates the cut-off level for WE-14 test assay. The * symbol refers to statistical difference between median values of controls *vs* each group. *PHEO*, pheochromocytoma; *PGL*, paraganglioma; *post-op*, post-operative patients; *preop*, preoperative patients. *ns*, not significant. ***, *p*<0.001; **, *p*<0.01. The numbers refer to the gene mutation as follow : *1*, SDHD ; *2*, SDHB ; *3*, NF1 ; *4*, RET ; *5*, VHL.

In healthy volunteers (n = 21), plasma concentrations of WE-14 ranged from 0.525–2.106 ng/ml with a median value of 0.689 ng/ml (Figure 2A, Table 1). The concentrations of WE-14 in preoperative patients (n = 37), irrespective of the localization (adrenal vs extra-adrenal), the status (benign vs malignant) or the sporadic vs hereditary nature of the tumor, ranged from 0.494–61.027 ng/ml with a median value of 3.695 ng/ml (Figure 2A, Table 1). Statistical analysis revealed that the median value of WE-14 concentrations was significantly higher in patients than in healthy volunteers (*p*<0.001). WE-14 levels were also measured in patients after surgical removal of the tumor (Figure 2C, n = 12). Most of these patients had elevated preoperative WE-14 concentrations that post-operatively returned to levels comparable to those of control subjects (Figure 2C, Table 1). In the cohort of 37 patients with chromaffin cell tumors, 26 had an adrenal localization of the tumor and 11 had an extra-adrenal neoplasm. Plasma concentrations of WE-14 were not significantly different between these two groups [3.204 (0.896–61.027) vs 5.484 (0.494–47.245) ng/ml, respectively] (Figure 2B, Table 1); while for both groups the WE-14 median values were significantly higher compared to healthy volunteers (*p*<0.001) (Figures 2A-B). The concentrations of WE-14 were not significantly different between patients with benign (n = 32) and malignant (n = 5) pheochromocytomas [3.776 (0.494–47.245) vs 3.078 (1.345–61.027) ng/ml, respectively] (Figure 2B, Table 1); however, median values for both tumor subtypes were significantly higher than for that of controls (*p*<0.001 for benign and *p*<0.05 for malignant tumors) (Figures 2A-B). Similarly, WE-14 concentrations were not significantly different in patients with sporadic pheochromocytomas (n = 24) compared to patients with hereditary tumors (n = 13) [4.224 (1.303–61.027) vs 2.542 (0.494–19.354) ng/ml, respectively] (Figure 2B, Table 1), but median values for both tumor groups were significantly higher than that in healthy volunteers (*p*<0.001, *p*<0.05 respectively) (Figures 2A-B).

**Table 1:** WE-14 plasma concentrations in healthy volunteers and patients with pheochromocytoma.

		WE-14 (ng/ml)			Sensitivity (%)						
		Median	Minimal value	Maximal value	WE-14	CgA	EM66	WE-14 +CgA	WE-14 +EM66	CgA +EM66	WE-14 +CgA+EM66
Controls	(n = 21)	0.689	0.525	2.106	-	-	-	-	-	-	-
Patients	(n = 37)	3.695^***^	0.494	61.027	83.8	83.8	83.8	91.9	91.9	89,2	94,6
Postoperative	(n = 12)	1.154	0.382	1.997	-	-	-	-	-	-	-
Pheochromocytoma	(n = 26)	3.204^***^	0.896	61.027	80,8	84,6	80,8	92,3	88,5	88,5	92,3
Paraganglioma	(n = 11)	5.484^***^	0.494	47.245	90,9	81,8	90,9	90,9	100	90,9	100
Benign	(n = 32)	3.776^***^	0.494	47.245	84,4	84,4	90,6	90,6	93,8	90,6	93,8
Malignant	(n = 5)	3.078*	1.345	61.027	80	80	40	100	80	80	100
Sporadic	(n = 24)	4.224^***^	1.303	61.027	91,7	95,8	87,5	100	95,8	95,8	100
Hereditary	(n = 13)	2.542*	0.494	19.354	69,2	61,5	76,9	76,9	84,6	76,9	84,6

Comparison of the diagnostic sensitivity of WE-14, CgA and EM66 assays.

*, median value of controls *vs* median value of patients, pheochromocytoma, paraganglioma, benign, malignant, sporadic or hereditary tumors. * *p*<0.05, *** *p*<0.001; *n*, number of individuals.

Table 1 summarizes the median, maximal and minimal values of WE-14 concentrations for controls and each group of patients. When an analysis was performed on a subgroup of 17 patients for which pheochromocytoma was surgically resected, no correlation could be found between tumor size and WE-14 levels (*r*  =  0.249) (data not shown).

### Plasma levels of CgA in patients with pheochromocytoma

**Figure 3:**
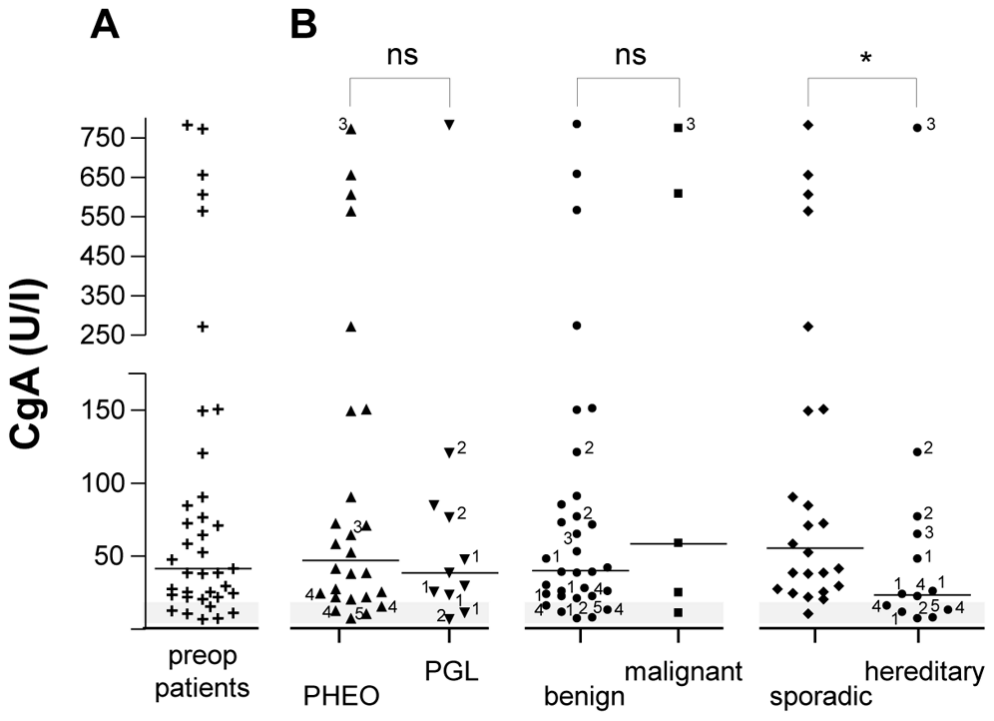
CgA levels in the plasma of patients with pheochromocytoma. (**A**) Scattergram of CgA concentrations in plasma samples of patients with pheochromocytoma before surgical removal of the tumor (preop, **+**, n = 37). (**B**) Distribution of CgA preoperative concentrations of patients reported in (A), depending on the adrenal (PHEO, ▴, n = 26) or extra-adrenal (PGL, ▾, n =  11) location of the tumor, the benign (•, n = 32) or malignant (▪, n = 5), and the sporadic (♦, n = 24) or hereditary (•, n = 13) nature of the neoplasms. The *grey zone* represents the cut-off level for the CgA test assay as indicated by the manufacturer. See legends of Figure 2 for other designations.

**Figure 4:**
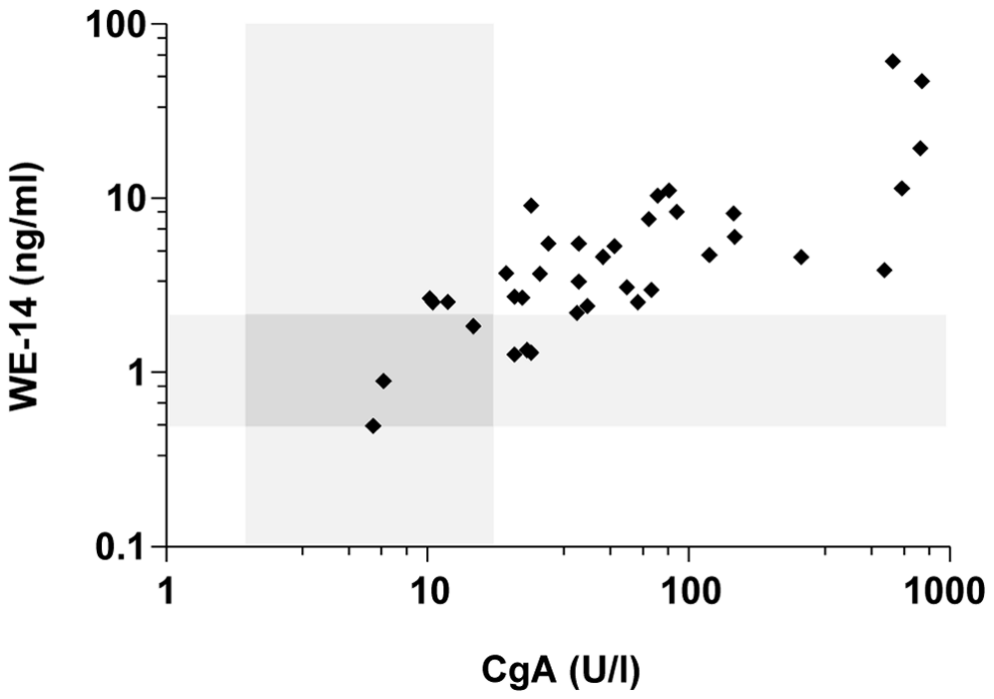
Correlation of plasma levels of WE-14 and CgA in patients with pheochromocytoma. The occurrence of the tumor leads to an elevation of plasma WE-14, which was significantly and positively correlated with the elevation of CgA (*r*  =  0.72, *p*<0.0001, n  =  37). The *grey zones* indicate the upper limits (cut-off values for the test assays) of WE-14 and CgA concentrations in healthy volunteers. Values included in these zones correspond to false-negative test results. *r,* Pearson correlation coefficient.

Plasma concentrations of CgA in preoperative patients (n = 37) ranged from 6-780 U/l with a median value of 41 U/l (Figure 3A). CgA concentrations were not significantly different between patients with pheochromocytoma or paraganglioma [46.5 (7-770) vs 38 (6-780) U/l, respectively] (Figure 3B). Similarly, CgA levels were not significantly different between patients with benign or malignant neoplasms [39.5 (6-780) vs 58 (10-770) U/l, respectively] (Figure 3B). In contrast, CgA concentrations were significantly higher in patients with sporadic compared to hereditary tumors [55 (10-780) vs 23 (6-770) U/l, respectively] (*p*<0.05) (Figure 3B). A positive correlation was found between CgA and WE-14 concentrations in the plasma of patients with pheochromocytoma in which high plasma levels of CgA are associated with high levels of WE-14 (*r*  =  0.717, *p*<0.001, n = 37) (Figure 4). In addition, except for malignant pheochromocytomas, WE-14 levels were positively correlated to CgA levels in each group of patients (data not shown). No correlation was found between tumor size and CgA levels (*r*  =  0.339) (data not shown).

### Plasma levels of EM66 in healthy volunteers and patients with pheochromocytoma

**Figure 5:**
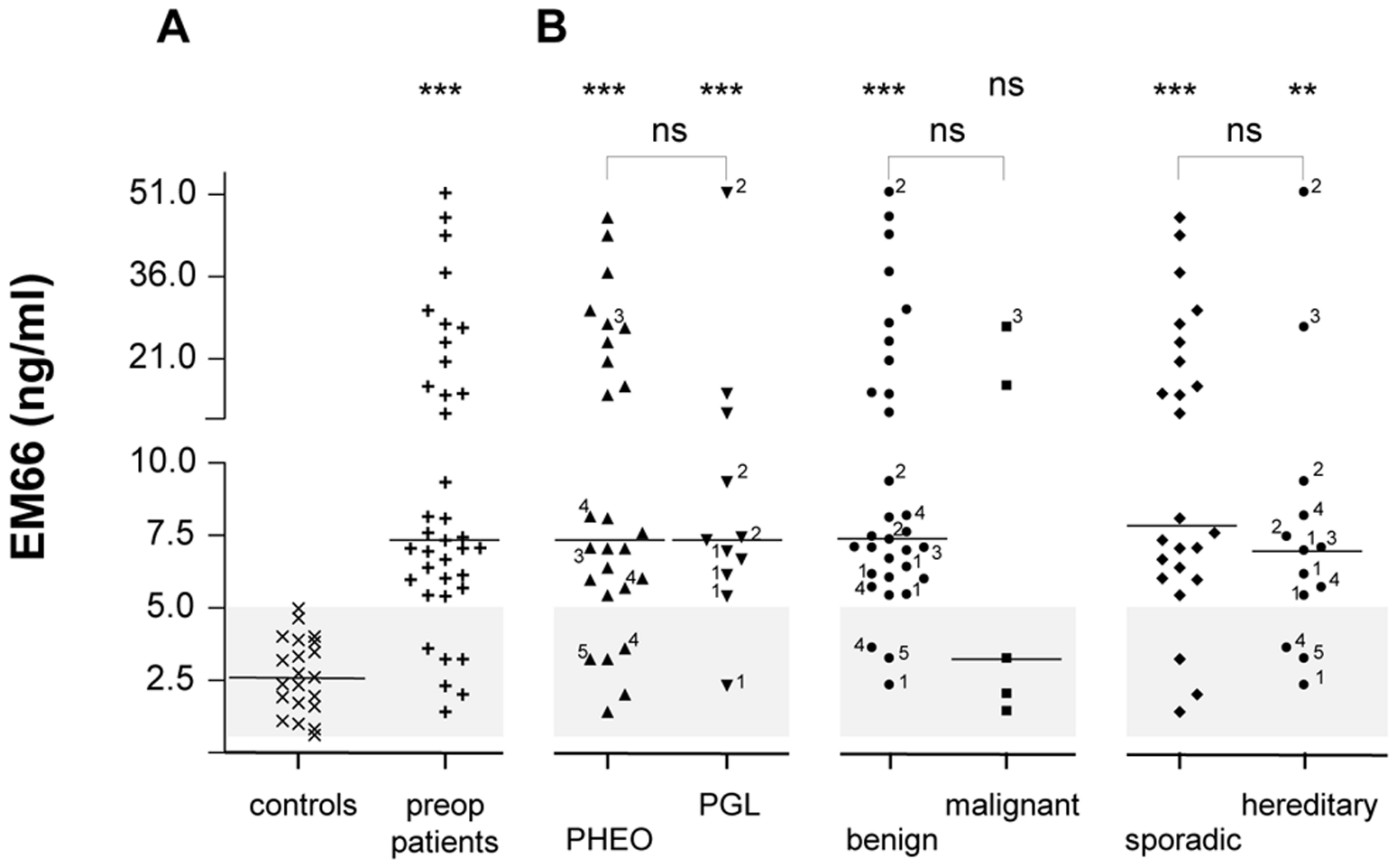
EM66 levels in the plasma of patients with pheochromocytoma. (**A**) Scattergram of EM66 concentrations in plasma samples of healthy volunteers (controls, **×**, n = 21), and patients with pheochromocytoma before surgical removal of the tumor (preop, **+**, n = 37). (**B**) Distribution of EM66 preoperative concentrations of patients reported in (A), depending on the adrenal (PHEO, ▴, n = 26) or extra-adrenal (PGL, ▾, n =  11) location of the tumor, the benign (•, n = 32) or malignant (▪, n = 5), and the sporadic (♦, n = 24) or hereditary (•, n = 13) nature of the neoplasm. The *grey zone* corresponds to the distribution of control values and indicates the cut-off level for EM66 test assay. See legends of Figure 2 for other designations.

In the plasma of healthy volunteers (n = 21), EM66 concentrations ranged from 0.615–4.953 ng/ml with a median value of 2.584 ng/ml (Figure 5A). The concentrations of EM66 in preoperative patients (n = 37) ranged from 1.411–51.260 ng/ml with a median value of 7.329 ng/ml (Figure 5A). Statistical analysis revealed that the median value of EM66 concentrations was significantly higher in patients than in healthy volunteers (*p*<0.001) (Figure 5A). EM66 concentrations were not significantly different between patients with pheochromocytomas or paraganglioma [7.323 (1.411–46.789) vs 7.329 (2.306–51.257) ng/ml, respectively] (Figure 5B), while for these two groups, the EM66 median values were significantly higher compared to healthy volunteers (*p*<0.001) (Figures 5A-B). Patients with benign pheochromocytomas did not show any significant difference in plasma EM66 concentrations compared to patients with malignant tumors [7.378 (2.306–51.257) vs 3.226 (1.411–26.611) ng/ml, respectively] (Figure 5B). Finally, EM66 concentrations were not significantly different between patients with sporadic or hereditary pheochromocytoma [7.829 (1.411–46.789) vs 6.950 (2.306–51.257) ng/ml, respectively] (Figure 5B), while for the two groups the EM66 median values were significantly higher than that in healthy volunteers (*p*<0.001, *p*<0.01, respectively) (Figures 5A-B). A positive correlation was found between WE-14 and EM66 or between CgA and EM66 concentrations in plasma of patients with pheochromocytoma (*r*  =  0.509 and *r*  =  0.470, respectively; *p*<0.01, n = 37) (data not shown). No correlation was found between tumor size and EM66 levels (*r*  =  0.286) (data not shown).

### Sensitivity of WE-14, CgA and EM66 test assays for the diagnosis of pheochromocytoma

The cut-off values for the WE-14 and EM66 test assays were defined as the maximal peptide concentration measured in healthy volunteers (2.106 ng/ml and 4.953 ng/ml, respectively). For CgA, the cut-off value (18 U/L) was that indicated by the manufacturer of the commercial kit. The sensitivity of WE-14 test assay was 83.8% in our total series of patients (n = 37), and ranged from 69.2% (hereditary tumors) to 91.7% (sporadic tumors), depending on the tumor subtype (Table 1). CgA and EM66 test assays showed identical diagnostic sensitivity to WE-14 (83.8%) in the group of 37 patients. Nevertheless, the sensitivities for these two markers varied between 61.5% (hereditary tumors) and 95.8% (sporadic tumors), and between 40% (malignant tumors) and 90.9% (paragangliomas), respectively (Table 1). For all patients (n = 37), whatever the test combination performed (WE-14 + CgA, WE-14 + EM66, or CgA + EM66), the sensitivity for the diagnosis of pheochromocytoma was increased (to approximately 90%) compared to each test alone. Moreover, the combination of the three test assays gave an even better sensitivity (about 95%) than that of two test assays. Table 1 summarizes the diagnostic sensitivity of the test assays for each group of patients. For instance, whatever the characteristic of the tumor, WE-14 measurement always increased the diagnostic sensitivity. Moreover, 100% sensitivity was reached when WE-14 and CgA tests were combined for the diagnosis of sporadic or malignant tumors, and when WE-14 and EM66 tests were used for the diagnosis of paragangliomas.

## Discussion

Using a cohort of patients with pheochromocytoma, we characterized the occurrence and evaluated the plasma levels of the WE-14 peptide. We also examined the relevance of combining the measurement of WE-14, CgA and EM66, in comparison to CgA alone, for the potential improvement of the diagnostic sensitivity for this tumor.

Previous studies have established that WE-14 is a distinct neuropeptide produced by cell-specific proteolysis of CgA in various neuronal and neuroendocrine tissues [40]. The early detection of WE-14 during ontogeny and the elevated concentrations detected in neuroendocrine neoplasms suggested that WE-14 could exert a physiological and/or pathophysiological role. WE-14 was initially isolated from a pheochromocytoma extract [34], and we have previously demonstrated that the peptide is present in chromaffin cells of the human fetal and adult adrenal medulla, and in pheochromocytoma [32], and is secreted from cultured pheochromocytes in primary cultures [33]. In the present study, HPLC analysis revealed that in the plasma of healthy volunteers as in normal adrenochromaffin cells, WE-14 immunoreactivity mainly corresponded to the 14-amino acid form of the peptide, suggesting that authentic WE-14 is released from neuroendocrine tissues into the circulation. Because WE-14 is present in pheochromocytes, this peptide could also be released into the circulation of patients with pheochromocytoma. Therefore, we characterized WE-14 in the plasma of such patients and compared its concentrations with those of healthy volunteers. In the 37 patients bearing the tumor, WE-14 was readily detectable in the plasma, and its preoperative levels were 5-fold higher than those of control subjects, suggesting that tumoral cells produce and release higher amounts of WE-14. In agreement with this hypothesis, we observed that after surgical removal of pheochromocytoma, plasma values of WE-14 concentrations were similar to those measured in control subjects. Interestingly, although our group of patients included adrenal or extra-adrenal tumors, familial or sporadic tumors, and benign or malignant tumor subtypes, they all showed significantly elevated levels of WE-14 compared to controls. Together, our data indicate that the measurement of WE-14 in plasma may represent a novel potential clinical tool for the diagnosis and follow-up of chromaffin cell tumors. Since only five malignant pheochromocytomas were included in the present study, no conclusion can be drawn concerning the value of WE-14 as a plasma marker of malignancy. It should be noted that the incidence of pheochromocytoma is only 2–8 cases per 1,000,000 subjects, and that malignant pheochromocytomas only represent about 10% of all pheochromocytomas. Further studies will be required to compare the concentration of WE-14 in patients with benign vs malignant tumors in larger cohorts, and to determine whether, in addition to its potential diagnostic usefulness for pheochromocytoma, WE-14 could also be used to evaluate the outcome of the disease.

Based on the cut-off value, we determined an overall 84% diagnostic sensitivity of the WE-14 test assay in our series of patients. However, we found a higher sensitivity (>90%) for the diagnosis of paraganglioma and sporadic tumors. Currently, CgA is widely used as a marker for pheochromocytoma and several CgA assays are commercially available [41]. Using samples from our present tumor cohort, we found that the sensitivity of the CgA test was identical to that of WE-14. Interestingly, the combination of the tests for the granin and its derived-peptide systematically offered a better sensitivity for all the tumor subtypes, since false-negative results were not obtained for the same patients with the two tests. A study by Stridsberg et al. [42] comparing three kits for plasma CgA measurements in various neuroendocrine tumors has revealed that the sensitivity of the different tests varied depending on the kit used. This may partly explain why the WE-14 RIA test could be used as a complementary tool. Moreover, plasma measurement of CgA may generate false-negative and false-positive test results for the diagnosis of neuroendocrine tumors [43]–[46]. Although we found a positive correlation between CgA and WE-14 concentrations in our cohort, there are patients who are positive for one marker but negative for the other. In patients with a negative CgA test but a positive WE-14 test, it is possible that the granin undergo complete processing in tumoral chromaffin cells leading to elevated WE-14 levels. For the patients with a negative WE-14 test but a positive CgA test, tumorigenesis may alter peptide processing or secretion resulting in WE-14 negative test results. The observation of false-negative results emphasizes the need to investigate the specificity of this peptide in order to improve the accuracy of the test for the diagnosis of pheochromocytoma and possibly other neuroendocrine tumors.

Whatever the test used and the characteristic of the tumor, at least one false-negative result was observed. The reason for the existence of these false-negative results remains unknown. Worthy of note, the patient with a VHL mutation had false-negative results for all three tests. Similarly, one patient with a RET mutation also had negative results on CgA, WE-14 and EM66 assays, while the two other patients with this gene mutation had at least one false-negative result. Further studies should be performed on a larger group of pheochromocytoma patients with a VHL or RET gene mutation to confirm these observations. Another interesting finding concerns the fact that CgA concentrations are significantly lower in patients with hereditary than sporadic tumors contrasting with the WE-14 and EM66 peptides which showed no variation, an observation that also deserves further investigation.

We previously demonstrated that EM66 is a sensitive plasma marker of pheochromocytoma [29]. Our present data indicate that the diagnostic sensitivity of the EM66 test assay, like that of CgA, is identical to that of WE-14. Furthermore, the EM66 test assay was systematically complementary to that of the CgA-derived peptide. For instance, the combination of the test assays of these two granin-derived peptides offered 100% sensitivity for the diagnosis of paragangliomas. This result is of particular interest since it has been reported that CgA has a very low diagnostic sensitivity for extra-adrenal chromaffin tumors [47]. Interestingly, all the tumors with a SDHB or SDHD mutation in our series corresponded to paragangliomas and, in this subgroup of patients, combined measurement of CgA, WE-14 and EM66 also provided 100% sensitivity. Collectively, our data show that combined CgA and granin-derived peptide test assays should possibly improve the diagnosis of pheochromocytoma. Combinatorial test assays could be achieved through development of multiplex immunoassays. Indeed, numerous studies have already shown the relevance of multiplex marker immunoassays [48]. Besides the benefit of this technology to simultaneously detect and quantify different analytes within one single small sample volume, it was shown that these assays may have a broader range and lower detection limit than the corresponding ELISA assays [49].

The current gold standard for diagnostic testing of pheochromocytoma relies on plasma or urine metanephrine measurement, which offers high sensitivity and specificity. However, these tests also have limitations [50]. For instance, the methodologies used to determine catecholamine metabolite measurement might differ depending on the clinical centers where this analysis is performed. In the present study, the 37 plasma samples were collected from three different centers where metanephrines were measured either from plasma or from urine. Therefore, we could not compare the results from the combined measurements of CgA, WE-14 and EM66 to the gold standard for the diagnosis of pheochromocytoma. However, we previously reported that, in a series of patients with pheochromocytoma, some of the patients had true positive plasma EM66 results and false-negative metanephrine results [29]. Besides false-negative results, false-positive results can also be generated when one measures metanephrines produced as a result of deficiency or pharmacological inhibition of monoamine oxidase (which leads to increased urinary deconjugated and plasma free metanephrines), or by medications such as tricyclic antidepressants (which account for up to 45% of false-positive elevation of plasma or urinary norepinephrine and normetanephrine). These observations indicate that while combined measurement of various forms of metanephrines offers a high diagnostic sensitivity and specificity, this biochemical test alone is obviously not sufficient for screening all pheochromocytoma and additional markers such as granins and their derived peptides are required to increase the diagnosis accuracy for this tumor.

In conclusion, we have shown that the CgA-derived peptide WE-14 represents a potential sensitive marker for the diagnosis and follow-up of pheochromocytoma. Currently, biochemical tests used for the diagnosis of pheochromocytoma include the measurement of plasma or urinary catecholamine metabolites (metanephrine, normetanephrine, methoxytyramine) and CgA assay. However, false-negative and false-positive results using biochemical tests remain a problem, leading to costly and time-consuming additional testing and imaging examinations [51], [52]. Therefore, while our study needs to be substantiated in a larger group of patients and confirmed prospectively in an independent series, our present results suggest that combined measurement of granins and their derived peptides, such as CgA, WE-14 and EM66, should accompany routine assays of metanephrine levels for the diagnosis of pheochromocytoma.

## References

[1] Fischer-Colbrie R, Laslop A, Kirchmair R (1995) Secretogranin II: molecular properties, regulation of biosynthesis and processing to the neuropeptide secretoneurin. Prog Neurobiol 46: 49–70. 7568909 10.1016/0301-0082(94)00060-u

[2] Winkler H, Fischer-Colbrie R (1992) The chromogranins A and B: the first 25 years and future perspectives. Neuroscience 49: 497–528. 1501763 10.1016/0306-4522(92)90222-N PMC7131462

[3] Helle KB (2004) The granin family of uniquely acidic proteins of the diffuse neuroendocrine system: comparative and functional aspects. Biol Rev Camb Philos Soc 79: 769–794. 15682870 10.1017/s146479310400644x

[4] Guerin M, Guillemot J, Thouennon E, Pierre A, El-Yamani FZ, et al (2010) Granins and their derived peptides in normal and tumoral chromaffin tissue: Implications for the diagnosis and prognosis of pheochromocytoma. Regul Pept 165: 21–29. 20600356 10.1016/j.regpep.2010.06.003

[5] Zhao E, Zhang D, Basak A, Trudeau VL (2009) New insights into granin-derived peptides: evolution and endocrine roles. Gen Comp Endocrinol 164: 161–174. 19523383 10.1016/j.ygcen.2009.01.011

[6] Rosa P, Gerdes HH (1994) The granin protein family: markers for neuroendocrine cells and tools for the diagnosis of neuroendocrine tumors. J Endocrinol Invest 17: 207–225. 8051343 10.1007/BF03347721

[7] Taupenot L, Harper KL, O'Connor DT (2003) The chromogranin-secretogranin family. N Engl J Med 348: 1134–1149. 12646671 10.1056/NEJMra021405

[8] Ferrari L, Seregni E, Bajetta E, Martinetti A, Bombardieri E (1999) The biological characteristics of chromogranin A and its role as a circulating marker in neuroendocrine tumours. Anticancer Res 19: 3415–3427. 10629629

[9] O'Connor DT, Deftos LJ (1986) Secretion of chromogranin A by peptide-producing endocrine neoplasms. N Engl J Med 314: 1145–1151. 3007986 10.1056/NEJM198605013141803

[10] Baudin E, Bidart JM, Bachelot A, Ducreux M, Elias D, et al (2001) Impact of chromogranin A measurement in the work-up of neuroendocrine tumors. Ann Oncol 12 Suppl 2: S79–82. 11762357 10.1093/annonc/12.suppl_2.s79

[11] Guignat L, Bidart JM, Nocera M, Comoy E, Schlumberger M, et al (2001) Chromogranin A and the alpha-subunit of glycoprotein hormones in medullary thyroid carcinoma and phaeochromocytoma. Br J Cancer 84: 808–812. 11259096 10.1054/bjoc.2000.1677 PMC2363821

[12] Cunningham RT, Pogue KM, Curry WJ, Johnston CF, Sloan JM, et al (1999) Immunostaining for vasostatin I distinguishes between ileal and lung carcinoids. J Pathol 187: 321–325. 10398086 10.1002/(SICI)1096-9896(199902)187:3<321::AID-PATH258>3.0.CO;2-9

[13] Sekiya K, Ghatei MA, Salahuddin MJ, Bishop AE, Hamid QA, et al (1989) Production of GAWK (chromogranin-B 420-493)-like immunoreactivity by endocrine tumors and its possible diagnostic value. J Clin Invest 83: 1834–1842. 2723061 10.1172/JCI114089 PMC303903

[14] Yasuda D, Iguchi H, Funakoshi A, Wakasugi H, Sekiya K, et al (1993) Comparison of plasma pancreastatin and GAWK concentrations, presumed processing products of chromogranin A and B, in plasma of patients with pancreatic islet cell tumors. Horm Metab Res 25: 593–595. 8288164 10.1055/s-2007-1002184

[15] Vieau D, Rojas-Miranda A, Verley JM, Lenne F, Bertagna X (1991) The secretory granule peptides 7B2 and CCB are sensitive biochemical markers of neuro-endocrine bronchial tumours in man. Clin Endocrinol (Oxf) 35: 319–325. 1752059 10.1111/j.1365-2265.1991.tb03543.x

[16] Eder U, Fischer-Colbrie R, Kogner P, Leitner B, Bjellerup P, et al (1998) Levels and molecular forms of chromogranins in human childhood neuroblastomas and ganglioneuromas. Neurosci Lett 253: 17–20. 9754794 10.1016/s0304-3940(98)00588-6

[17] Ischia R, Gasser RW, Fischer-Colbrie R, Eder U, Pagani A, et al (2000) Levels and molecular properties of secretoneurin-immunoreactivity in the serum and urine of control and neuroendocrine tumor patients. J Clin Endocrinol Metab 85: 355–360. 10634410 10.1210/jcem.85.1.6314

[18] Ischia R, Hobisch A, Bauer R, Weiss U, Gasser RW, et al (2000) Elevated levels of serum secretoneurin in patients with therapy resistant carcinoma of the prostate. J Urol 163: 1161–1164 discussion 1164–1165. 10737487

[19] Neumann HP, Bausch B, McWhinney SR, Bender BU, Gimm O, et al (2002) Germ-line mutations in nonsyndromic pheochromocytoma. N Engl J Med 346: 1459–1466. 12000816 10.1056/NEJMoa020152

[20] Karasek D, Frysak Z, Pacak K (2010) Genetic testing for pheochromocytoma. Curr Hypertens Rep 12: 456–464. 20938758 10.1007/s11906-010-0151-1 PMC3061287

[21] Thouennon E, Pierre A, guillemot J, Yon L, Eisenhofer G, et al (2009) Genetic markers for the diagnosis and prognosis of pheochromocytoma. Expert Rev Endocrinol Metab 4: 45–52. 10.1586/17446651.4.1.45 30934373

[22] Vicha A, Musil Z, Pacak K (2013) Genetics of pheochromocytoma and paraganglioma syndromes: new advances and future treatment options. Curr Opin Endocrinol Diabetes Obes 20: 186–191. 23481210 10.1097/MED.0b013e32835fcc45 PMC4711348

[23] Amar L, Bertherat J, Baudin E, Ajzenberg C, Bressac-de Paillerets B, et al (2005) Genetic testing in pheochromocytoma or functional paraganglioma. J Clin Oncol 23: 8812–8818. 16314641 10.1200/JCO.2005.03.1484

[24] Andersen KF, Altaf R, Krarup-Hansen A, Kromann-Andersen B, Horn T, et al (2011) Malignant pheochromocytomas and paragangliomas - the importance of a multidisciplinary approach. Cancer Treat Rev 37: 111–119. 20675056 10.1016/j.ctrv.2010.07.002

[25] Anouar Y, Jegou S, Alexandre D, Lihrmann I, Conlon JM, et al (1996) Molecular cloning of frog secretogranin II reveals the occurrence of several highly conserved potential regulatory peptides. FEBS Lett 394: 295–299. 8830661 10.1016/0014-5793(96)00976-3

[26] Anouar Y, Desmoucelles C, Yon L, Leprince J, Breault L, et al (1998) Identification of a novel secretogranin II-derived peptide (SgII_187–252_) in adult and fetal human adrenal glands using antibodies raised against the human recombinant peptide. J Clin Endocrinol Metab 83: 2944–2951. 9709974 10.1210/jcem.83.8.5009

[27] Guillemot J, Ait-Ali D, Turquier V, Montero-Hadjadje M, Fournier A, et al (2006) Involvement of multiple signaling pathways in PACAP-induced EM66 secretion from chromaffin cells. Regul Pept 137: 79–88. 16963134 10.1016/j.regpep.2006.04.023

[28] Montero-Hadjadje M, Pelletier G, Yon L, Li S, Guillemot J, et al (2003) Biochemical characterization and immunocytochemical localization of EM66, a novel peptide derived from secretogranin II, in the rat pituitary and adrenal glands. J Histochem Cytochem 51: 1083–1095. 12871990 10.1177/002215540305100812

[29] Guillemot J, Anouar Y, Montero-Hadjadje M, Grouzmann E, Grumolato L, et al (2006) Circulating EM66 is a highly sensitive marker for the diagnosis and follow-up of pheochromocytoma. Int J Cancer 118: 2003–2012. 16287097 10.1002/ijc.21571

[30] Guillemot J, Thouennon E, Guerin M, Vallet-Erdtmann V, Ravni A, et al (2012) Differential expression and processing of secretogranin II in relation to the status of pheochromocytoma: implications for the production of the tumoral marker EM66. J Mol Endocrinol 48: 115–127. 22217803 10.1530/JME-11-0077

[31] Yon L, Guillemot J, Montero-Hadjadje M, Grumolato L, Leprince J, et al (2003) Identification of the secretogranin II-derived peptide EM66 in pheochromocytomas as a potential marker for discriminating benign versus malignant tumors. J Clin Endocrinol Metab 88: 2579–2585. 12788858 10.1210/jc.2002-021748

[32] Montero-Hadjadje M, Vaudry H, Turquier V, Leprince J, Do Rego JL, et al (2002) Localization and characterization of evolutionarily conserved chromogranin A-derived peptides in the rat and human pituitary and adrenal glands. Cell Tissue Res 310: 223–236. 12397377 10.1007/s00441-002-0625-9

[33] Guillemot J, Compagnon P, Cartier D, Thouennon E, Bastard C, et al (2009) Metoclopramide stimulates catecholamine- and granin-derived peptide secretion from pheochromocytoma cells through activation of serotonin type 4 (5-HT4) receptors. Endocr Relat Cancer 16: 281–290. 18948374 10.1677/ERC-08-0190

[34] Conlon JM, Hamberger B, Grimelius L (1992) Isolation of peptides arising from the specific posttranslational processing of chromogranin A and chromogranin B from human pheochromocytoma tissue. Peptides 13: 639–644. 1437706 10.1016/0196-9781(92)90167-2

[35] Curry WJ, Shaw C, Johnston CF, Thim L, Buchanan KD (1992) Isolation and primary structure of a novel chromogranin A-derived peptide, WE-14, from a human midgut carcinoid tumour. FEBS Lett 301: 319–321. 1577173 10.1016/0014-5793(92)80266-j

[36] Gleeson CM, Curry WJ, Johnston CF, Buchanan KD (1996) Occurrence of WE-14 and chromogranin A-derived peptides in tissues of the human and bovine gastro-entero-pancreatic system and in human neuroendocrine neoplasia. J Endocrinol 151: 409–420. 8994386 10.1677/joe.0.1510409

[37] Hanna FW, Ardill JE, Johnston CF, Cunningham RT, Curry WJ, et al (1997) Regulatory peptides and other neuroendocrine markers in medullary carcinoma of the thyroid. J Endocrinol 152: 275–281. 9071985 10.1677/joe.0.1520275

[38] Heaney AP, Curry WJ, Pogue KM, Armstrong VL, Mirakhur M, et al (2000) Immunohistochemical evaluation of the post-translational processing of chromogranin A in human pituitary adenomas. Pituitary 3: 67–75. 11141698 10.1023/a:1009949623054

[39] Leprince J, Gandolfo P, Thoumas JL, Patte C, Fauchere JL, et al (1998) Structure-activity relationships of a series of analogues of the octadecaneuropeptide ODN on calcium mobilization in rat astrocytes. J Med Chem 41: 4433–4438. 9804683 10.1021/jm980275d

[40] Curry WJ, Quinn JG, Brockbank S, Miller V, McVicar C, et al (2004) Comparative Studies of the Chromogranin A-Derived Neuropeptide WE-14. Curr Med Chem 4: 213–219.

[41] d'Herbomez M, Forzy G, Bauters C, Tierny C, Pigny P, et al (2007) An analysis of the biochemical diagnosis of 66 pheochromocytomas. Eur J Endocrinol 156: 569–575. 17468193 10.1530/EJE-06-0640

[42] Stridsberg M, Eriksson B, Oberg K, Janson ET (2003) A comparison between three commercial kits for chromogranin A measurements. J Endocrinol 177: 337–341. 12740022 10.1677/joe.0.1770337

[43] Sanduleanu S, De Bruine A, Stridsberg M, Jonkers D, Biemond I, et al (2001) Serum chromogranin A as a screening test for gastric enterochromaffin-like cell hyperplasia during acid-suppressive therapy. Eur J Clin Invest 31: 802–811. 11589723 10.1046/j.1365-2362.2001.00890.x

[44] Stridsberg M, Oberg K, Li Q, Engstrom U, Lundqvist G (1995) Measurements of chromogranin A, chromogranin B (secretogranin I), chromogranin C (secretogranin II) and pancreastatin in plasma and urine from patients with carcinoid tumours and endocrine pancreatic tumours. J Endocrinol 144: 49–59. 7891024 10.1677/joe.0.1440049

[45] Borch K, Stridsberg M, Burman P, Rehfeld JF (1997) Basal chromogranin A and gastrin concentrations in circulation correlate to endocrine cell proliferation in type-A gastritis. Scand J Gastroenterol 32: 198–202. 9085454 10.3109/00365529709000194

[46] Spadaro A, Ajello A, Morace C, Zirilli A, D'Arrigo G, et al (2005) Serum chromogranin-A in hepatocellular carcinoma: diagnostic utility and limits. World J Gastroenterol 11: 1987–1990. 15800991 10.3748/wjg.v11.i13.1987 PMC4305722

[47] Nobels FR, Kwekkeboom DJ, Coopmans W, Schoenmakers CH, Lindemans J, et al (1997) Chromogranin A as serum marker for neuroendocrine neoplasia: comparison with neuron-specific enolase and the alpha-subunit of glycoprotein hormones. J Clin Endocrinol Metab 82: 2622–2628. 9253344 10.1210/jcem.82.8.4145

[48] Loo BM, Marniemi J, Jula A (2011) Evaluation of multiplex immunoassays, used for determination of adiponectin, resistin, leptin, and ghrelin from human blood samples, in comparison to ELISA assays. Scand J Clin Lab Invest 71: 221–226. 21288160 10.3109/00365513.2011.554996

[49] Kim YW, Bae SM, Kim IW, Liu HB, Bang HJ, et al (2012) Multiplexed bead-based immunoassay of four serum biomarkers for diagnosis of ovarian cancer. Oncol Rep 28: 585–591. 22641176 10.3892/or.2012.1829

[50] Därr R, Pamporaki C, Peitzsch M, Miehle K, Prejbisz A, et al (2013) Biochemical diagnosis of phaeochromocytoma using plasma-free normetanephrine, metanephrine and methoxytyramine: importance of supine sampling under fasting conditions. Clin Endocrinol doi: 10.1111/cen.12327 10.1111/cen.12327 24102244

[51] Neumann HP (2002) Imaging vs biochemical testing for pheochromocytoma. Jama 288: 314–315; author reply 315. 10.1001/jama.288.3.314 12117391

[52] Sawka AM, Gafni A, Thabane L, Young WF Jr (2004) The economic implications of three biochemical screening algorithms for pheochromocytoma. J Clin Endocrinol Metab 89: 2859–2866. 15181069 10.1210/jc.2003-031127

